# Epoxy-Containing Ionic Liquids with Tunable Functionality

**DOI:** 10.3390/molecules24142591

**Published:** 2019-07-17

**Authors:** Tetsuya Tsuda, Kazuki Iwasaki, Kohei Kumagai, Susumu Kuwabata

**Affiliations:** Department of Applied Chemistry, Graduate School of Engineering, Osaka University, 2-1 Yamada-oka, Suita, Osaka 565-0871, Japan

**Keywords:** ionic liquid, epoxy group, physicochemical property, carbon dioxide, functionality change

## Abstract

New types of ionic liquids (ILs) with an epoxy group on a piperidinium-type cation were successfully synthesized by the simple anion exchange reaction of a solid 1-allyl-1-(oxiran-2-ylmethyl)piperidinium bromide, which was designed in this study. Unfortunately, the physicochemical properties, e.g., viscosity and ionic conductivity, of the ILs were inferior to those of common ILs such as 1-ethyl-3-methylimidazolium tetrafluoroborate ([C_2_mim][BF_4_]) and 1-butyl-3-methylimidazolium bis(trifluoromethanesulfonyl)amide ([C_4_mim][Tf_2_N]). However, the resulting ILs are of great interest as reaction intermediates: For example, the epoxy group on the cation could react with various reagents, including CO_2_. Consequently, the modification of the cation structure in the ILs was possible. This is particularly interesting because it is very difficult to modify commonly used ILs. The approach established in this article will provide a favorable synthetic route for creating novel functional ILs in the future.

## 1. Introduction

An ionic liquid (IL) is a liquid salt consisting entirely of cations and anions. ILs are becoming popular in science and technology because of their interesting features, such as their negligible vapor pressure, incombustibility, wide electrochemical window, relatively high ionic conductivity, and antistatic properties [1,2,3], which make them unique liquids. Therefore, ILs are currently considered to be functional liquid materials [4,5,6] and are used in a wide range of areas, including as nonvolatile and fire-retardant electrolytes for electrochemical devices [2,3,4,7,8,9], green solvents for organic synthesis processes [1], and reaction media for nanomaterial fabrication processes [4,10,11]. In addition, novel analytical techniques with ILs have also been proposed [4,12,13,14,15].

Because ILs are designable liquid salts, a great number of IL families have been reported to date [1,2,3,16,17]. Ionic liquids are normally prepared using the anion exchange reaction between an organic salt with a simple halide ion (such as Br^−^ or Cl^−^) and an alkali metal salt with a target anion [1,2,3]. The organic cation in the salt can be readily designed using synthetic chemistry techniques. Basic skeletons for the typical cations are 1,3-dialkylimidazolium, *N*-alkylpyridinium, *N*,*N*-dialkylpyrrolidinium, *N*,*N*-piperidinium, linear alkylammonium, and linear alkylphosphonium. Various side-chains can be added to the skeletons. Many counter anions, including [AlCl_4_]^−^, [BF_4_]^−^, [PF_6_]^−^, [CF_3_SO_3_]^−^, [N(SO_2_CF_3_)_2_]^−^, [N(CN)_2_]^−^, and [(FH)*_n_*F]^−^ (1 ≤ *n* ≤ 3), are known. Because of the diversity of ionic species, the physicochemical properties can be controlled to a certain extent. For example, the thermal stability of ILs with fluoroanions is better than with monoatomic halide anions that have stronger nucleophilicity [18,19]. This suggests that we can control the thermal stability, as well as the other physicochemical properties, by changing the anionic species in the ILs. 

Although ILs are very useful solvents and liquid materials, it is difficult to modify the ionic species in ILs further using synthetic chemistry techniques, except for polymerization reactions via the vinyl group [20,21] and CO_2_ addition reactions via the amino group [22]. If modifications were possible using a convenient reaction scheme, the options for tailoring IL structures would be broadened. Previously, we established a synthetic scheme for producing ILs with a propylene carbonate group [23]. In this study, referring to the synthetic scheme, novel ILs with an epoxy group, which is highly reactive, were designed, and their physicochemical properties were examined. The aim of this study was to clarify whether the ILs produced could react with various reagents and be modified through these reactions. 

## 2. Results and Discussion

### 2.1. Thermophysical Properties

**Scheme 1:**
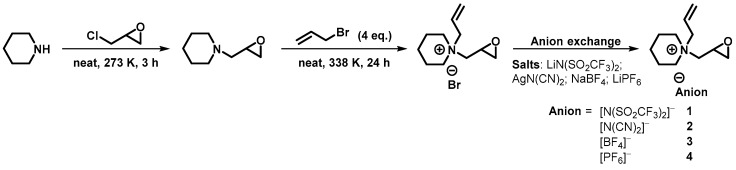
Synthesis of 1-allyl-1-(oxiran-2-ylmethyl)piperidinium-based organic salts.

**Figure 1:**
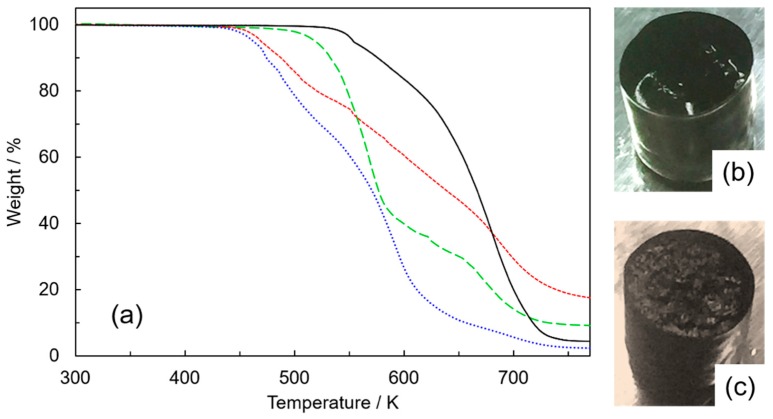
(**a**) Results of TGA measurements of the 1-allyl-1-(oxiran-2-ylmethyl)piperidinium-based organic salts **1**–**4**: (―) **1**, (- - -) **2**, (• • •) **3**, and (‒ ‒ ‒) **4**. The rate of temperature increase was 5 K min^−1^. Photographs of ionic liquid (IL) **2** after thermogravimetry measurements under (**b**) nitrogen and (**c**) air atmosphere conditions.

Using the procedures depicted in Scheme 1, four 1-allyl-1-(oxiran-2-ylmethyl)piperidinium-based organic salts (**1**–**4**) were successfully obtained. At 293 K, **1** and **2** were fluids: That is, they were ILs. In contrast, **3** and **4** were quite solid. Figure 1 shows the results of thermogravimetric analysis (TGA) measurements of the resultant organic salts under a nitrogen atmosphere. It is generally considered that ILs containing the bis(trifluoromethanesulfonyl)amide anion ([N(SO_2_CF_3_)_2_]^‒^) show better thermal stability than those with the dicyanamide anion ([N(CN)_2_]^‒^) [2,18,19]. As expected, the thermal stability of IL **1** having [N(SO_2_CF_3_)_2_]^‒^ was superior to that of **2** having [N(CN)_2_]^‒^. The thermal decomposition of **3** and **4** was initiated at 460–470 K. Interestingly, the residue for **2** was greater than ~17 wt %. Ionic liquids with [N(CN)_2_]^‒^ are often exploited to synthesize nitrogen-doped functional carbon materials because [N(CN)_2_]^‒^ is a multiple cross-linkable component for polytriazine network formation with a high carbonization yield [24,25,26]. When the TGA measurements of **2** were conducted in an air atmosphere, the residue exceeded 25 wt %. The moisture in the air may have been involved in the increase in the carbonaceous residue because the epoxide-opening reaction of the 1-allyl-1-(oxiran-2-ylmethyl)piperidinium cation occurred in the presence of water, as discussed in detail later. Figure 1b,c shows photographs of **2** placed on the Al pan after the thermogravimetry measurements under a nitrogen and air atmosphere, respectively. Graphitic foam was produced only under the latter condition, suggesting that **2** is a potential precursor for synthesizing functional carbon materials. 

**Figure 2:**
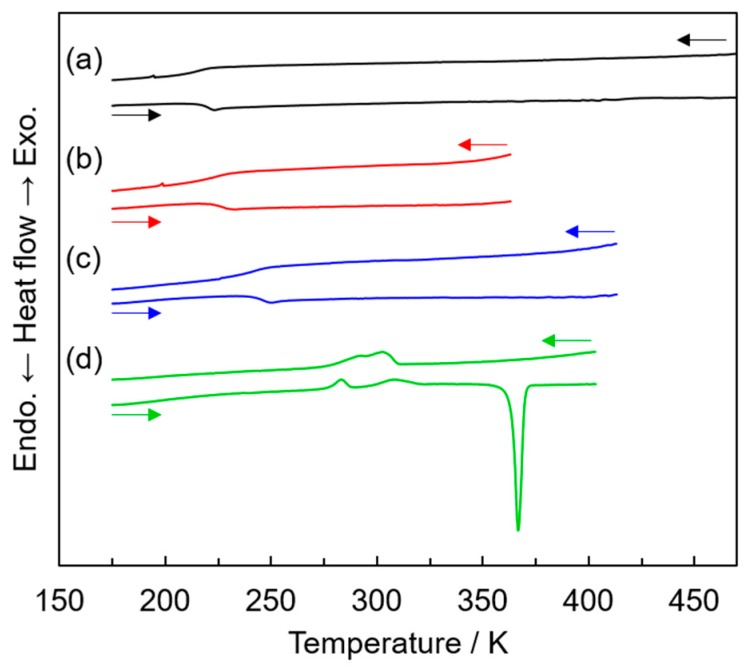
Differential scanning calorimetry curves of the 1-allyl-1-(oxiran-2-ylmethyl)piperidinium-based organic salts: (**a**) **1**, (**b**) **2**, (**c**) **3**, and (**d**) **4**.

The differential scanning calorimetry (DSC) curves of the organic salts **1**–**4** are shown in Figure 2. Only **4** showed different phase transition behavior, i.e., very small exothermic peaks for the crystallization at ~279 K and the crystalline phase transition at ~299 K. Because it is not uncommon for ILs to show similar behavior, these phase changes could be governed by the very slow dynamics in **4** [27,28]. In addition to this behavior, a clear endothermic peak for the melting process at 363 K appeared in the DSC curve during the heating process. The other organic salts (**1**–**3**) showed only glass transitions at corresponding temperatures (*T*_g_) of 221–241 K, like most ILs. All the thermophysical properties are summarized in Table 1, along with other solution properties. 

### 2.2. Solution Properties

**Figure 3:**
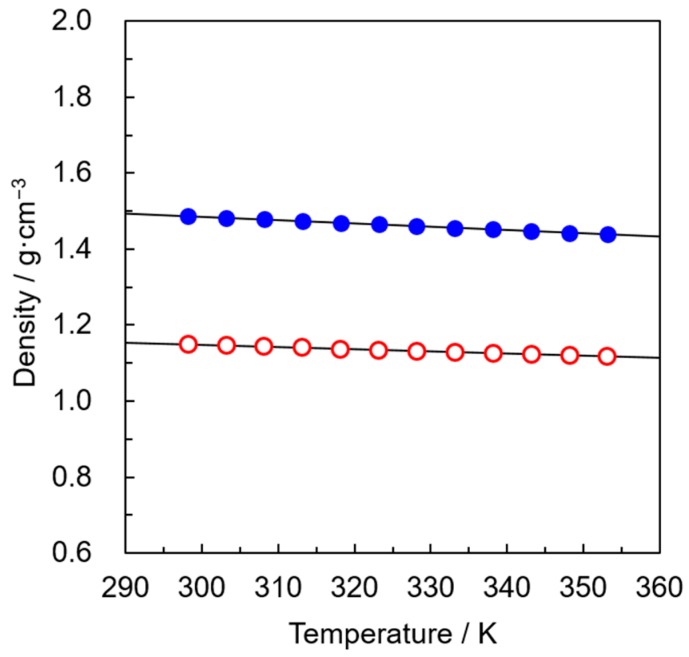
Temperature dependence of density of 1-allyl-1-(oxiran-2-ylmethyl)piperidinium-based ILs (●) **1** and (○) **2**.

The obtained organic salts **1**–**4** were divided into two categories: **1** and **4** were hydrophobic and **2** and **3** were hydrophilic. The temperature dependence of the density, *ρ* (g·cm^−3^), of ILs **1** and **2** is shown in Figure 3. A linear dependence with absolute temperature was obtained. The solid lines were derived from calculation using the least-squares method. The density is expressed as a function of temperature:

*ρ* = *a* + *bT* (1)

where *a*, *b*, and *T* are the density at 0 K (g·cm^−3^), the coefficient of volume expansion (g·cm^−3^·K^−1^), and the absolute temperature (K), respectively. The fitting parameters estimated from the least-squares method are summarized in Table 2. All the density data for **1** were larger than those of **2**. It is generally considered that it is the anionic species in the ILs that greatly influence the density if the cation is the same, and the density roughly correlates with the anionic formula weight [29] or the anionic volume [30,31]. Our results suggest that the density of the 1-allyl-1-(oxiran-2-ylmethyl)piperidinium cation-based ILs can be controlled by the anionic formula weight. 

**Figure 4:**
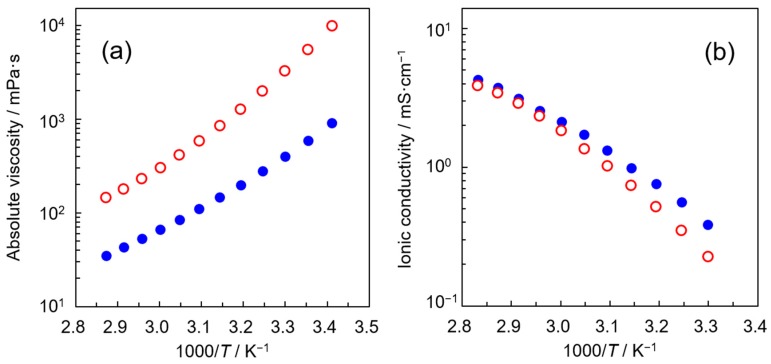
Arrhenius plots of (**a**) absolute viscosity and (**b**) ionic conductivity of 1-allyl-1-(oxiran-2-ylmethyl)piperidinium-based ILs (●) **1** and (○) **2**.

Arrhenius plots of the absolute viscosity, *η* (mPa·s), and ionic conductivity, *σ* (mS·cm^−1^), for **1** and **2** are shown in Figure 4a,b, respectively. The obtained plots show nonlinear behavior because these ILs were glass-forming liquids, as described above. In such cases, the data should be represented by the Vogel–Tammann–Fulcher (VTF) equation to obtain precise physicochemical insight into the transport behavior [32,33]. However, the activation energies of the absolute viscosity and ionic conductivity could be roughly determined by a simple approach using the linear part of the Arrhenius plots. The results are given in Table 3. At temperatures over 333 K, the activation energies for **1** were smaller than for **2** because there were fewer ionic interactions.

### 2.3. Carboxylation and Decarboxylation Reactions

**Scheme 2:**
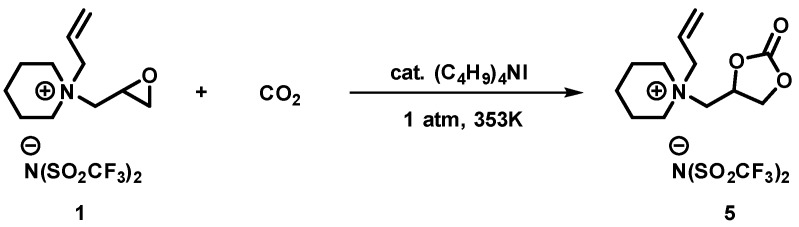
Synthesis of IL **5** through a carbonylation reaction using IL **1** and gaseous CO_2_.

**Figure 5:**
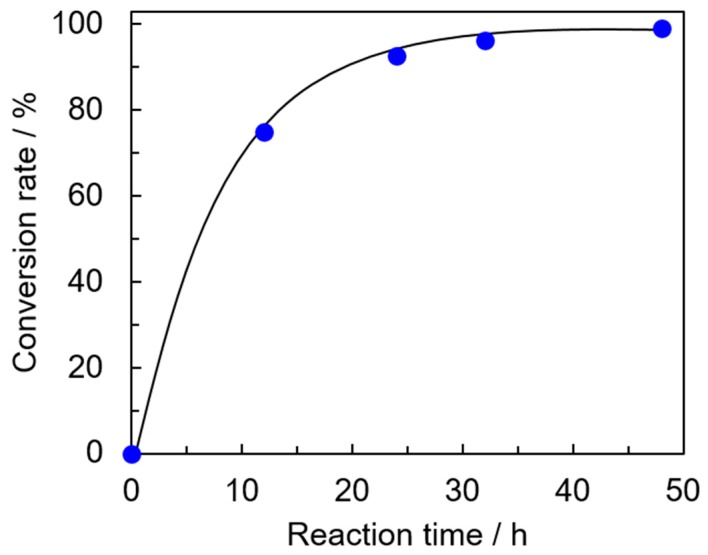
IL **1** conversion rate via a carbonylation reaction using gaseous CO_2_.

Carboxylation and decarboxylation reactions using the chemically reactive epoxy group on the 1-allyl-1-(oxiran-2-ylmethyl)piperidinium cation were attempted for ILs **1** and **2** because, if the reactions were reversible, we could employ them as functional ILs for capturing CO_2_ both chemically and physically. The carboxylation reaction of **1**, which had a lower viscosity than **2**, was carried out following Scheme 2 using tetrabutylammonium iodide as a catalyst and atmospheric CO_2_ gas. The reaction temperature was 353 K. After 24 h, the conversion rate was over 92%, and finally, the cations fully reacted with CO_2_ (Figure 5). The decarboxylation reaction of the resulting IL **5**, through a simple heating process, was also examined. However, it was very difficult to obtain the 1-allyl-1-(oxiran-2-ylmethyl)piperidinium cation without a side reaction, and the weight loss corresponding to CO_2_ release, which was identified by NMR and mass spectroscopy, was initiated at temperatures over ~473 K.

### 2.4. Epoxide-Opening Reactions

**Scheme 3:**
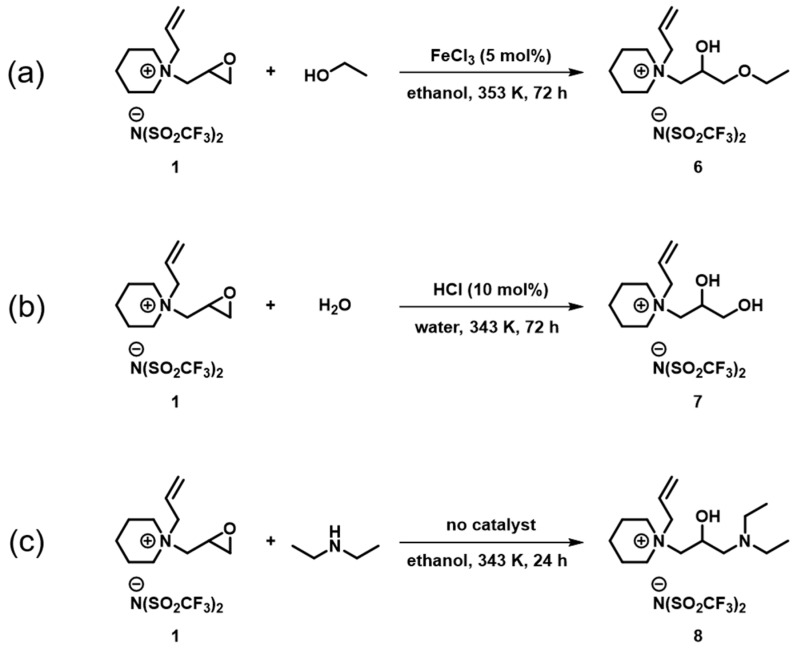
Synthesis of ILs **6**–**8** through the epoxide-opening reactions of IL **1**. The reaction reagents were (**a**) ethanol, (**b**) water, and (**c**) diethylamine.

As is well known, epoxy groups on neutral molecules can be changed to various chemical structures via epoxide-opening reactions with several reagents [34]. However, except for network polymer formation, there have been no reports of the epoxide-opening reaction on the organic cation in ILs [35]. To examine these reactions, three reagents were tested: Ethanol, water, and diethylamine. All the reactions were conducted at temperatures over 343 K. After each chemical process, IL **1** was converted into a novel IL, **6**–**8**, as shown in Scheme 3, even though the modification of the organic cation in an IL is usually a very challenging task. Thus, we found that the use of the 1-allyl-1-(oxiran-2-ylmethyl)piperidinium cation-based ILs enabled the further modification of the cation in the IL. 

## 3. Materials and Methods 

### 3.1. Synthesis of 1-(Oxiran-2-ylmethyl)piperidine

1-(Oxiran-2-ylmethyl)piperidine was synthesized using piperidine (Sigma-Aldrich, Inc., St. Louis, MO, USA) and epichlorohydrin (Tokyo Chemical Industry Co., Ltd., Tokyo, Japan) and the following procedure. An aqueous solution (3 mL) of 1000 mmol of epichlorohydrin was added to cooled piperidine (1000 mmol), and the mixture was stirred for 1 h at 273 K, followed by further agitation at 293 K for 30 min. Then, 30% NaOH aq was added to the mixture with stirring for 3 h. After the reaction was complete, the product was extracted with diethyl ether and rinsed with ultrapure water several times. The combined organics were dried over MgSO_4_. The solvent was evaporated, and the resulting light yellow crude product was purified with a Kugelrohr distillation apparatus. The colorless final product was confirmed by ^1^H NMR, ^13^C NMR, and mass spectrometry. ^1^H NMR (400 MHz, DMSO-*d*_6_) δ_H_ 1.36 (m, 2H, *J* = 5.7 Hz), 1.49 (m, 4H, *J* = 5.7 Hz), 2.15 (dd, 1H, *J* = 13.2 Hz, 6.4 Hz), 2.34–2.41 (m, 5H), 2.53 (dd, 1H, *J* = 13.6 Hz, 4.0 Hz), 2.67 (t, 1H, *J* = 5.2 Hz), 2.97 (m, 1H, *J* = 3.3 Hz). ^13^C-NMR (100 MHz, DMSO-*d*^6^) δ_C_ 23.9 (s), 25.5 (s), 44.2 (s), 49.8 (s), 54.4 (s), 61.1 (s). High-resolution mass spectrometry (HRMS) (electron ionization (EI)), *m/z*: calcd for C_8_H_15_NO 141.1154 [M]^+^; found 141.1156. 

### 3.2. Synthesis of 1-Allyl-1-(oxiran-2-ylmethyl)piperidinium Bromide

1-Allyl-1-(oxiran-2-ylmethyl)piperidinium bromide was synthesized using allylbromide (Kishida Chemical Co., Ltd., Osaka, Japan) and 1-(oxiran-2-ylmethyl)piperidine prepared through the above procedure. First, 500 mmol of 1-(oxiran-2-ylmethyl)piperidine was added to the allylbromide (4 eq), and the mixture was stirred for 24 h at 338 K. As the reaction proceeded, a clay-like precipitate was formed. The resulting product was separated by decantation and rinsed with diethyl ether three times. After drying under reduced pressure for 4 h at 338 K, the target salt was obtained as a white solid. The final product was confirmed by ^1^H NMR, ^13^C NMR, and mass spectrometry. ^1^H NMR (400 MHz, DMSO-*d*^6^) δ_H_ 1.54 (m, 2H, *J* = 6.0 Hz), 1.82 (m, 4H, *J* = 5.2 Hz), 2.72 (dd, 1H, *J* = 2.2 Hz, 5.0 Hz), 2.92 (t, 1H, *J* = 4.8 Hz), 3.22 (dd, 1H, *J* = 14.2 Hz, 8.6 Hz), 3.31–3.50 (m, 4H), 3.53–3.56 (m, 1H), 3.84 (d, 1H, *J* = 13.6 Hz), 4.11–4.26 (m, 2H), 5.61–5.73 (m, 2H), 6.00–6.11 (m, 1H). ^13^C NMR (100 MHz, DMSO-*d*_6_) δ_C_ 18.9 (s), 19.0 (s), 20.6 (s), 44.1 (s), 44.3 (s), 58.4 (s), 58.5 (s), 60.8 (s), 61.2 (s), 125.4 (s), 127.6 (s). HRMS (fast atom bombardment (FAB)), *m/z*: calcd for C_11_H_20_NO^+^ 182.1539 [M − Br]^+^; found 182.1543. 

### 3.3. General Synthetic Procedure for Organic Salts with Epoxy Moieties

The 1-allyl-1-(oxiran-2-ylmethyl)piperidinium-based organic salts 1-allyl-1-(oxiran-2-ylmethyl)piperidinium bis(trifluoromethanesulfonyl)amide, **1**, 1-allyl-1-(oxiran-2-ylmethyl)piperidinium dicyanamide, **2**, 1-allyl-1-(oxiran-2-ylmethyl)piperidinium tetrafluoroborate, **3**, and 1-allyl-1-(oxiran-2-ylmethyl)piperidinium hexafluorophosphate, **4**, were synthesized by the anion exchange reaction between 1-allyl-1-(oxiran-2-ylmethyl)piperidinium bromide and the appropriate inorganic salt containing the target anionic species. Those inorganic salts were lithium bis(trifluoromethanesulfonyl)amide (Morita Chemical Industries Co., Ltd., Osaka, Japan), silver dicyanamide, sodium tetrafluoroborate (Sigma-Aldrich, Inc., St. Louis, MO, USA), and lithium hexafluorophosphate (Wako Pure Chemical Industries, Ltd., Osaka, Japan) (Scheme 1). The synthetic protocols are described below in detail. The inorganic salts (30 mmol) were added to dichloromethane (50 mL) with 1-allyl-1-(oxiran-2-ylmethyl)piperidinium bromide (30 mmol), and the mixtures were stirred for 24 h at ambient temperature. After the reaction, the mixtures were filtered to remove the precipitated byproduct, and the filtrate was condensed under vacuum. The crude product extracted by dichloromethane was rinsed with ultrapure water several times to remove the unreacted reagent and byproduct completely. The organic layer was concentrated in vacuo. The resultant salt was dried at 338 K under vacuum for 24 h. The final product was confirmed by ^1^H NMR, ^13^C NMR, and mass spectrometry.

#### 3.3.1. 1-Allyl-1-(oxiran-2-ylmethyl)piperidinium bis(trifluoromethanesulfonyl)amide (**1**) 

^1^H NMR (400 MHz, DMSO-*d*^6^) δ_H_ 1.55 (m, 2H, *J* = 6.1 Hz), 1.82 (m, 4H, *J* = 5.4 Hz), 2.71 (dd, 1H, *J* = 2.4 Hz, 4.8 Hz), 2.92 (t, 1H, *J* = 4.8 Hz), 3.19 (dd, 1H, *J* = 14.8 Hz, 8.8 Hz), 3.28–3.47 (m, 4H), 3.51–3.55 (m, 1H), 3.81 (dd, 1H, *J* = 14.8 Hz, 1.2 Hz), 4.08–4.23 (m, 2H), 5.61–5.72 (m, 2H), 5.98–6.10 (m, 1H). ^13^C NMR (100 MHz, DMSO-*d*^6^) δ_C_ 18.9 (s), 19.0 (s), 20.6 (s), 44.0 (s), 44.3 (s), 58.5 (s), 58.6 (s), 60.9 (s), 61.2 (s), 119.5 (q, J = 320.1 Hz), 125.4 (s), 127.7 (s). HRMS (FAB), *m/z*: calcd for C_24_H_40_N_3_O_6_S_2_F_6_^+^ 644.2257 [M + C_11_H_20_NO]^+^; found 644.2264.

#### 3.3.2. 1-Allyl-1-(oxiran-2-ylmethyl)piperidinium dicyanamide (**2**)

^1^H NMR (400 MHz, DMSO-*d*^6^) δ_H_ 1.55 (m, 2H, *J* = 6.2 Hz), 1.82 (m, 4H, *J* = 5.6 Hz), 2.71 (dd, 1H, *J* = 2.8 Hz, 4.8 Hz), 2.92 (t, 1H, *J* = 4.8 Hz), 3.19 (dd, 1H, *J* = 14.8 Hz, 8.8 Hz), 3.28–3.47 (m, 4H), 3.51–3.55 (m, 1H), 3.81 (dd, 1H, *J* = 14.8 Hz, 1.6 Hz), 4.09–4.23 (m, 2H), 5.61–5.72 (m, 2H), 5.99–6.12 (m, 1H). HRMS (FAB), *m/z*: calcd for C_24_H_40_N_5_O_2_^+^ 430.3177 [M + C_11_H_20_NO]^+^; found 430.3186.

#### 3.3.3. 1-Allyl-1-(oxiran-2-ylmethyl)piperidinium tetrafluoroborate (**3**)

^1^H NMR (400 MHz, DMSO-*d*^6^) δ_H_ 1.55 (m, 2H, *J* = 6.1 Hz), 1.82 (m, 4H, *J* = 5.8 Hz), 2.72 (dd, 1H, *J* = 2.6 Hz, 5.0 Hz), 2.92 (t, 1H, *J* = 4.8 Hz), 3.20 (dd, 1H, *J* = 14.6 Hz, 8.6 Hz), 3.29–3.48 (m, 4H), 3.51–3.56 (m, 1H), 3.82 (dd, 1H, *J* = 14.8 Hz, 2.0 Hz), 4.10–4.24 (m, 2H), 5.61–5.72 (m, 2H), 5.99–6.10 (m, 1H). HRMS (FAB), *m/z*: calcd for C_22_H_40_N_2_O_2_BF_4_^+^ 451.3113 [M + C_11_H_20_NO]^+^; found 451.3125.

#### 3.3.4. 1-Allyl-1-(oxiran-2-ylmethyl)piperidinium hexafluorophosphate (**4**)

^1^H NMR (400 MHz, DMSO-*d*^6^) δ_H_ 1.55 (m, 2H, *J* = 6.1 Hz), 1.82 (m, 4H, *J* = 5.8 Hz), 2.71 (dd, 1H, *J* = 2.2 Hz, 5.0 Hz), 2.92 (t, 1H, *J* = 4.8 Hz), 3.18 (dd, 1H, *J* = 14.8 Hz, 8.8 Hz), 3.27–3.47 (m, 4H), 3.51–3.55 (m, 1H), 3.81 (dd, 1H, *J* = 14.8 Hz, 2.0 Hz), 4.08–4.22 (m, 2H), 5.61–5.72 (m, 2H), 5.99–6.10 (m, 1H). HRMS (FAB), *m/z*: calcd for C_22_H_40_N_2_O_2_PF_6_^+^ 509.2726 [M + C_11_H_20_NO]^+^; found 509.2733.

### 3.4. Physicochemical Property Measurements 

TGA experiments were performed using a Bruker TG-DTA2000SA (Billeria, MA, USA). The sample was placed in an aluminum pan without an aluminum top, and the pan was heated from room temperature to 773 K at a rate of 1 or 5 K min^−1^ under the flow of dry nitrogen gas. The thermal degradation temperature was determined from the 1% weight loss point of the TG curve. DSC measurements were conducted using a Bruker DSC3100SA (Billeria, MA). The sample was sealed in an aluminum pan with an aluminum top. The sealed pan was heated and cooled at a rate of 5 K min^−1^. The glass-transition temperature and melting point were obtained from the DSC curve of the second heating process. These values were estimated by the tangential intersection method near the temperature at which a phase transformation occurred. These two instruments were controlled with a Bruker MTC1000SA (Billeria, MA) workstation utilizing Bruker WS003 software (Billeria, MA). All specimens for these measurements were prepared in an argon-filled glove box (Vacuum Atmospheres Co., Omni-Lab, Hawthome, CA) O_2_ and H_2_O < 1 ppm).

Density measurements were conducted using a Kyoto Electronics Manufacturing DA-640 resonant frequency oscillation density/specific gravity meter (Kyoto, Japan) in a range of 298–353 K. Viscosity was measured using a Kyoto Electronics Manufacturing EMS-1000 electromagnetically spinning viscometer (Kyoto, Japan) in a range of 293–348 K. An ionic conductivity measurement was carried out by electrochemical impedance spectroscopy with a Princeton Applied Research VersaSTAT4 potentiostat/galvanostat (Oak Ridge, TN, USA) using a calibrated two-electrode cell composed of two platinum discs in a range of 303–353 K. The cell constant was determined using three concentrations (0.1, 0.01, and 0.001 M) of a standard aqueous KCl solution at 298 K. All measurements except for viscosity were conducted in an argon-filled glove box, as described above. The viscosity was measured using a special airtight cell for the electromagnetically spinning viscometer.

### 3.5. Carbonylation of Ionic Liquid **1** Using Gaseous CO_2_

The chemical reaction between IL **1** and CO_2_ was carried out with tetrabutylammonium iodide (Sigma-Aldrich, Inc., St. Louis, MO, USA) as a catalyst. First, 0.05 eq of tetrabutylammonium iodide was added to 3 mmol of **1**. Under a CO_2_ gas atmosphere (1 atm), the mixture was stirred for 48 h at 353 K (Scheme 2). To follow the reaction progress, ^1^H NMR measurements were made on samples taken at 12, 24, 32, and 48 h. After the reaction, the mixture was rinsed with ultrapure water and extracted with dichloromethane several times. The organic layer was concentrated in vacuo. The product was confirmed by ^1^H NMR and mass spectrometry.

#### 1-Allyl-1-[(2-oxo-1,3-dioxolan-4-yl)methyl]piperidinium bis(trifluoromethanesulfonyl)amide (**5**)

^1^H NMR (400 MHz, DMSO-*d*^6^) δ_H_ (400 MHz, DMSO-*d*^6^) 1.49–1.61 (m, 2H), 1.83 (m, 4H, *J* = 5.6 Hz), 3.27–3.46 (m, 4H), 3.61 (d, 1H, *J* = 15.2 Hz), 4.04–4.18 (m, 4H), 4.70 (t, 1H, *J* = 8.8 Hz), 5.47 (q, 1H, *J* = 8.4 Hz), 5.61–5.70 (m, 2H), 5.97–6.09 (m, 1H). HRMS (FAB), *m/z*: calcd for C_16_H_20_N_3_O_11_F_12_S_4_^−^ 785.9795 [M + N(SO_2_CF_3_)_2_]^−^; found 785.9814.

### 3.6. Epoxide-Opening Reaction of Ionic Liquid **1** with Several Reagents 

The details of the epoxide-opening reactions conducted in this study are described below.

#### 3.6.1. 1-Allyl-1-(3-ethoxy-2-hydroxypropyl)piperidinium bis(trifluoromethanesulfonyl)amide (**6**)

The chemical reaction between IL **1** and ethanol (Wako Pure Chemical Industries, Ltd., Osaka, Japan) was performed with anhydrous FeCl_3_ (Wako Pure Chemical Industries, Ltd., Osaka, Japan) as a catalyst. Anhydrous FeCl_3_ (5 mol %) was added to 1 mmol of IL **1** in 1 mL of ethanol. The mixture was stirred for 72 h at 353 K in an airtight test tube under an argon atmosphere (Scheme 3a). After the reaction, the condensed mixture extracted by dichloromethane was rinsed with ultrapure water several times. The organic layer was concentrated in vacuo. The product was confirmed by mass spectrometry. HRMS (FAB), *m/z*: calcd for C_28_H_52_F_6_N_3_O_8_S_2_^+^ 736.3095 [M + C_13_H_26_NO_2_]^+^; found 736.3089. 

#### 3.6.2. 1-Allyl-1-(2,3-dihydroxypropyl)piperidinium bis(trifluoromethanesulfonyl)amide (**7**)

The chemical reaction between IL **1** and water was performed with HCl (Wako Pure Chemical Industries, Ltd., Osaka, Japan) as a catalyst. First, 1 mmol of IL **1** was added to a 0.1-M aqueous HCl solution. The mixture was stirred for 72 h at 353 K in an airtight test tube under an air atmosphere (Scheme 3b). After the reaction was complete, the extra water and HCl were evaporated from the mixture. To remove the residual water, the mixture was dried thoroughly in vacuo. The product was confirmed by mass spectrometry. HRMS (FAB), *m/z*: calcd for C_15_H_22_F_12_N_3_O_10_S_4_^−^ 760.0002 [M + N(SO_2_CF_3_)_2_]^−^; found 759.9997.

#### 3.6.3. 1-Allyl-1-(3-(diethylamino)-2-hydroxypropyl)piperidinium bis(trifluoromethanesulfonyl)amide (**8**)

The chemical reaction between IL **1** and diethylamine (Wako Pure Chemical Industries, Ltd., Osaka, Japan) was performed without any catalyst. First, 2 mmol of diethylamine was added to 1 mmol of IL **1** in 1 mL of ethanol. The mixture was stirred for 24 h at 343 K in an airtight test tube in an air atmosphere (Scheme 3c). After the reaction was complete, the extra diethylamine and ethanol were evaporated from the mixture. The residual solvent was removed from the mixture by drying thoroughly in vacuo. The product was confirmed by mass spectrometry. HRMS (FAB), *m/z*: calcd for C_32_H_62_F_6_N_5_O_6_S_2_^+^ 790.4040 [M + C_15_H_31_N_2_O]^+^; found 790.4048.

## 4. Conclusions

Four 1-allyl-1-(oxiran-2-ylmethyl)piperidinium-based organic salts having an epoxy group on the piperidinium cation were successfully produced by synthetic chemistry techniques. When [N(SO_2_CF_3_)_2_]^−^ and [N(CN)_2_]^−^ were present in the salts, the compounds were anhydrous liquid salts at 298 K, i.e., ILs, but their transport properties were inferior to most [C_2_mim]^+^-based ILs. However, the ILs offer a significant advantage compared to other IL systems reported to date: The epoxy group on the cation could react with various reagents. This means that the chemical structure of the epoxy-containing ILs can be modified by the further chemical synthesis step and that other novel ILs can be created from the ILs. This finding reveals a useful approach for designing new functional ILs and will contribute to the further development of IL science and technology. 

## Figures and Tables

**Table 1:** Physicochemical properties of the 1-allyl-1-(oxiran-2-ylmethyl)piperidinium-based organic salts at 298 K.

Entry	FW *^a^*	*T*_m_*^b^* /K	*T*_g_*^c^* /K	*T*_d_*^d^*/K	*d ^e^*/g∙cm^-3^	*η ^f^*/mPa∙s	*σ ^g^* /mS∙cm^−1^	Solubility in Water
**1**	462.4	None	221.6	531.4	1.487	909	0.387	Insoluble
**2**	248.3	None	220.9	447.3	1.149	9890	0.228	Soluble
**3**	269.1	None	240.9	456.9	-	-	-	Soluble
**4**	327.3	363.5	None	471.1	-	-	-	Insoluble

*^a^* Formula weight. *^b^* Melting point. *^c^* Glass-transition point. *^d^* Thermal degradation temperature at 1 wt % loss. *^e^* Density. *^f^* Viscosity. *^g^* Ionic conductivity at 303 K.

**Table 2:** Fitted parameters for the density of the 1-allyl-1-(oxiran-2-ylmethyl)piperidinium-based ILs.

Entry	*a*/g·cm^−3^	*b* × 10^4^/g·cm^−3^·K^−1^	|*R*|
**1**	1.751	−8.834	>0.9999
**2**	1.321	−5.773	>0.9999

**Table 3:** Activation energies for the 1-allyl-1-(oxiran-2-ylmethyl)piperidinium-based ILs determined from the Arrhenius plots for absolute viscosity and ionic conductivity.

Entry	Activation Energy/kJ∙mol^−1^	
	Absolute Viscosity	Ionic Conductivity
**1**	41.7	34.8
**2**	50.2	36.8
